# IgG immune complex-induced acute lung injury is ameliorated by cAMP *via* down-regulation of C/EBP- and AP-1-mediated transcriptions

**DOI:** 10.1186/s12950-023-00359-6

**Published:** 2023-10-20

**Authors:** Chunguang Yan, Jing Chen, Huifang Tang, Chunmin Deng, Qi Zhang, Ximo Wang

**Affiliations:** 1 Department of Pathogenic Biology and Immunology, Medical School of Southeast University, Nanjing, 210009 China; 2 Jiangsu Provincial Key Laboratory of Critical Care Medicine, Zhongda Hospital of Southeast University, Nanjing, 210009 China; 3 Tianjin Key Laboratory of Acute Abdomen Disease Associated Organ Injury and ITCWM Repair, Institute of Integrative Medicine for Acute Abdominal Diseases, Integrated Chinese and Western Medicine Hospital, Tianjin University, Tianjin, 300100 China; 4 Zhejiang Respiratory Drugs Research Laboratory of the State Food and Drug Administration of China, School of Medicine, Zhejiang University, Hangzhou, 310058 Zhejiang China; 5 Department of Clinical Laboratory Medicine, Suzhou Science and Technology Town Hospital, Suzhou Hospital Affiliated to Nanjing Medical University, Suzhou, 215153 China

**Keywords:** Acute lung injury, cAMP, PKA, MAPK, AP-1, C/EBP

## Abstract

**Background:**

Acute lung injury (ALI) and its more severe form, acute respiratory distress syndrome (ARDS) are life threatening pulmonary diseases, and we are now lack of effective therapeutic methods. Inflammatory responses are essential for initiating ALI/ARDS. Thus, ameliorating inflammatory reaction might be beneficial for treatment of the disease. There are increasing data that phosphodiesterase-4 (PDE4)-selective inhibitors, which may elevate cellular cyclic adenosine 3′, 5′-monophosphate (cAMP) level, could suppress inflammation. However, whether they could be used to treat IgG immune complex (IgG-IC)-associated ALI has not been determined.

**Methods:**

ALI is induced by treating mice with airway deposition of IgG immune complexes. Cellular cAMP concentrations are elevated by treating mice or macrophages with Rolipram/Roflumilast. The degree of pulmonary injury is reflected by lung permeability, leukocyte accumulation, histological change and expressions of pro-inflammatory mediators. 6-Bnz-cAMP and H-89 are used to regulate protein kinase A (PKA) activity, and 8-pCPT-2′-O-Me-cAMP is applied to activate exchange proteins directly activated by cAMP (Epac). Gene expressions are analyzed by real-time PCR, ELISA or Western blot. CCAAT/enhancer binding protein (C/EBP) and activation protein 1 (AP-1) transcription activities are estimated by measuring the luciferase productions.

**Results:**

IgG-IC-induced ALI is attenuated by the PDE4-selective inhibitor, which is due to reduced expressions of cytokine and chemokines. Interestingly, we find that cAMP downstream effector molecule PKA but not Epac is involved in negative regulation of IgG-IC-mediated pro-inflammatory mediators’ productions. Mechanistically, activation of cAMP-PKA signal axis leads to inactivation of MAPK pathway, resulting in a decrease in C/EBP- and AP-1-mediated transcriptions of pro-inflammatory mediators.

**Conclusions:**

Our data demonstrate, for the first time, that cAMP-PKA signal is involved in down-regulation of IgG-IC-associated inflammatory responses *via* down-regulating MAPK activation, which is critical for transcriptional activities of C/EBP and AP-1. Collectively, our experiments provide theoretical base for the potential application of PDE4-selective inhibitor to clinic for treatment of IgG-IC-related acute lung injury.

**Supplementary Information:**

The online version contains supplementary material available at 10.1186/s12950-023-00359-6.

## Background

Acute lung injury (ALI) and acute respiratory distress syndrome (ARDS) are important problems in human beings [1]. Breakthroughs have been made in therapeutic methods, however, the morbidity and mortality of ALI/ARDS remain high [1]. Thus, we should develop more effective treatments according to currently identified pathogenesis. Pulmonary deposition of IgG immune complexes, which has been detected in patients suffering from ALI/ARDS, is associated with poor outcomes [2–4]. In the rodent model, intratracheal formation of IgG-IC is firstly sensed by macrophages expressing high level of Fcγ receptors [5, 6]. Activated macrophages then produce a variety of pro-inflammatory mediators including cytokines and chemokines, resulting in activation of endothelial cells and neutrophils [6–8]. Subsequently, pulmonary accumulation of polymorphonuclear cells is observed [8]. Finally, under the joint action of activated immune-related cells, lung epithelial and endothelial barriers are destroyed. Collectively, inflammatory responses triggered by activated macrophages are essential for initiating ALI/ARDS. Thus, attenuating inflammation might be beneficial for treatment of ALI/ARDS.

Cyclic adenosine 3′, 5′-monophosphate (cAMP) is one of the most important cellular second messengers with versatile functions, including cell proliferation, survival, migration and activation [9, 10]. Cellular cAMP level is elevated once adenylyl cyclases (AC), which can convert ATP to cAMP, is activated by upstream proteins such as G protein-coupled receptors [11]. Then downstream events, including activation of protein kinases A (PKA) which is a heterotetramer consisting of two catalytic subunits and two regulatory subunits, or Epac (exchange proteins directly activated by cAMP), could be observed [12–14]. Of note, cellular concentrations of cAMP are negatively regulated by PDEs (cyclic nucleotide phosphodiesterases) that hydrolyze the substrate to AMP [15]. PDE4 family members, composed of PDE4A, PDE4B, PDE4C and PDE4D, are mainly expressed by leukocytes and, therefore, are of particularly critical for inflammatory reactions [16]. By now, a vast collection of inhibitors targeting the PDE4 enzymatic activity have been developed. More importantly, Roflumilast, a PDE4-selective inhibitor, has been applied in clinic to treat severe COPD (chronic obstructive pulmonary disease) patients with exacerbation [17].

The anti-inflammatory role of cAMP and its downstream molecules have been widely demonstrated in different kinds of diseases and animal models. However, its effect on IgG-IC-induced inflammatory responses remains unknown. In this study, we find that inhibition of PDE4 activity reduces IgG-IC-induced generation of cytokine and chemokines in macrophages and mouse lungs, leading to alleviation of acute lung injury. Previous study has demonstrated that cAMP downstream target Epac but not PKA inhibits inflammatory responses in LPS-treated macrophages and lungs [18]. In contrast, PKA but not Epac is involved in transduction of anti-inflammatory signal in IgG-IC-induced inflammatory response. It have been wildly demonstrated that phosphorylation of p38 MAPK at Thr180/Tyr182 [19], phosphorylation of ERK1/2 at Thr202/Tyr204 [20], and phosphorylation of JNK at Ser63/Ser73 [21] promote acute lung injury. We, for the first time, demonstrate that cAMP-PKA signal suppresses activation of p38 MAPK, ERK1/2 and JNK, leading to a decrease in AP-1- and C/EBP-mediated transcriptions. Taken together, our results provide theoretical basis for application of the PDE4 inhibitor in clinic to treat patients with IgG-IC-associated acute lung injury.

## Materials and methods

### Cell Culture and reagents

RAW264.7 cells and HEK293 cells are obtained from ATCC, and maintained in DMEM (Dulbecco′s modified Eagle′s medium) supplemented with 10% fetal bovine serum (FBS) from Gibico. BSA (bovine serum albumin) and anti-BSA antibody (α-BSA) are obtained from Invitrogen and MP Biomedicals, respectively. Dimethyl sulfoxide (DMSO) is obtained from Sigma-Aldrich. Rolipram and Roflumilast are obtained from Cayman Chemical and APExBIO, respectively. 6-Bnz-cAMP (PKA agonist) and 8-pCPT-2′-O-Me-cAMP (Epac agonist) are obtained from Biolog Life Science Institute. PKA inhibitor H-89 is obtained from Beyotime Biotechnology. ELISA kits for measuring cAMP, TNF-α, MIP-1α, MIP-1β, MIP-2 and KC are all obtained from R&D Systems. ELISA kit for measuring mouse albumin is obtained from Bethyl Laboratories.

### IgG-IC-induced Acute Lung Injury [22–25]

Eight to twelve-week-old male mice (C57BL/6) are used in the study. All these pathogen-free mice are obtained from Model Animal Research Center of Nanjing University (Nanjing, China). The animal protocol of the study is approved by Experimental Ethical Committee of Southeast University. All methods are carried out in accordance with Southeast University guidelines for the use of live animals. Mice are anesthetized with intraperitoneal injection of 0.2 ml of 1.5% sodium pentobarbital. 100 µg of anti-BSA IgG dissolved in 40 µl of PBS is instilled intratracheally during inspiration. Immediately after airway injection of anti-BSA, 1 mg of BSA in 0.2 ml of PBS is injected through tail vein. Negative control mice receive intratracheal administration of anti-BSA. Unless otherwise indicated, 4 h later, mice are exsanguinated, and the lung circulation is flushed though the lung artery with 1ml of PBS. Then the animal lungs are surgically dissected for the following experiments. In the model, typical pulmonary inflammatory injury is observed 4 h after airway deposition of IgG-IC as mentioned by other groups and our previous data [22, 23, 26–28]. So, when constructing the model, we refer to these published papers. In the experiment, animal deaths are not induced by IgG-IC treatment.

### Measurement of lung MPO (myeloperoxidase) Activity [22–24, 29]

To evaluate lung MPO activity, lungs harvested from mice are immediately frozen in liquid nitrogen. Then whole pulmonary tissues are homogenized in 50 mmol/L potassium phosphate buffer including 0.5% hexadecyltrimethylammonium bromide and 5 mmol/L EDTA, which is followed by sonication and centrifuge. 10 µl of recovered supernatants are then mixed with 100 mmol/L potassium phosphate buffer including 167 µg/ml *o*-dianisidine dihydrochloride and 1.5 mol/L H_2_O_2_ in 96-well plates. MPO activity is determined by measurement of the optical density change at 450 nm over 3 min using a 96-well plate reader.

### Analysis of Bronchoalveolar Lavage (BAL) Fluids [22–25]

4 h after initiation of IgG-IC-induced acute lung injury, the thorax is opened and 1 ml of ice-cold, sterile PBS is instilled into the lung through a tracheal incision. Then lung BAL fluids are harvested. Mouse albumin, TNF-α, MIP-1α, MIP-1β, MIP-2 and KC are measured by ELISA according to the protocols provided by the manufacturers. The permeability index is calculated by dividing the albumin level in the IgG-IC-injured lung by that in the control lung from the same type of mouse, and the permeability index of control lung is set to 1. Total white blood cells are harvested by centrifuging BAL fluids at 3000 rpm for 5 min, and are counted using hemocytometers. To obtain neutrophil and macrophage counts, BAL fluid is processed by cytospin onto microscope slides. Then the cells are stained with the Wright’s dye solution. A total of 400 cells including neutrophils and macrophages per slide are counted in randomly selected high-powered fields (×1000). So, the percentages of neutrophils and macrophages could be calculated. Finally, the total number of neutrophils and macrophages in BAL fluids are evaluated.

### Pulmonary H&E staining

Four hours after airway deposition of IgG immune complex. The lungs are harvested and fixed in 4% formaldehyde. Then the fixed tissues are embedded in paraffin and cut into 4-µm sections. Finally, the tissue sections are stained with H&E (haematoxylin and eosin), and observed by microscopy.

### Isolation of RNA and RT-qPCR assay

Total cellular RNAs are isolated by using Trizol from Invitrogen, and cDNAs are obtained *via* reverse transcription. ChamQ SYBR Color qPCR Master Mix (Low ROX Premixed) purchased from Vazyme is used to analyze expressions of TNF-α, MIP-1α and MIP-1β according to the following protocol: denaturation at 95 °C for 30 s which is followed by 40 cycles of 95 °C for 10 s and 60 °C for 30 s. The used primers are listed as follows: TNF-α, 5′ primer, 5′-CGT CAG CCG ATT TGC TAT CT-3′ and 3′ primer, 5′-CGG ACT CCG CAA AGT CTA AG-3′; MIP-1α, 5′ primer, 5′ -ATG AAG GTC TCC ACC ACT GC-3 ′ and 3′ primer, 5′ -CCC AGG TCT CTT TGG AGT CA-3′; MIP-1β, 5′ primer, 5′ -tct gcc ctc tct ctc ctc tt-3 ′ and 3′ primer, 5′ -atg tac tca gtg acc cag gg-3′.

### Western blot assay

For Western blot assay, cells are lysed in RIPA buffer (Beyotime Biotechnology, China) after PBS washing. Sonication is applied to remove the viscosity of the lysates. Protein concentrations are determined by Enhanced BCA Protein Assay Kit purchased from Beyotime Biotechnology. 40 µg of proteins are electrophoresed on SDS-PAGE gels (Beyotime Biotechnology, China), and transferred to polyvinylidene difluoride membranes obtained from Millipore. Non-specific binding is blocked by incubating the membranes with 5% nonfat milk at room temperature for an hour. The membranes are incubated with the corresponding primary antibody at 4 °C for 12 h. After three washes, the membranes are incubated with the HRP (horseradish peroxidase)-conjugated secondary antibody at room temperature for an hour. Finally, the membranes are developed by using enhanced chemiluminescence Kit (Thermo Fisher Scientific). Immunoblotting is performed with primary antibodies against p-p38 MAPK (Thr180/Tyr182, Cell Signaling Technology), p-ERK1/2 (Thr202/Tyr204, Cell Signaling Technology), p-JNK (Ser63/Ser73, Santa Cruz), and GAPDH (Proteintech), respectively. GAPDH is used as the loading control in the experiment. All HRP-conjugated secondary antibodies are purchased from Proteintech. Biostep™ Prestained Protein Marker is purchased from Tanon. The prestained protein ladder is a mixture of ten proteins spanning 11 to 180 KD.

### Luciferase assay

HEK293/RAW264.7 cells are transfected with AP-1 Luc (Beyotime Biotechnology) plus TK (Promega) or C/EBP Luc (Promega) plus TK by using Fugene®6 from Promega. One day later, HEK293 cells are treated with DMSO or Rolipram (10 µM) for 6 h. RAW264.7 cells are pre-treated with DMSO or Rolipram (10 µM ) for 1 h. Then the cells are incubated with or without 100 µg/ml of IgG-IC for 6 h. The cells are lysed and subjected to luciferase analysis by using Dual-Luciferase Reporter Assay System from Promega. Firefly luciferase activity is first normalized to that of the renilla luciferase driven by thymidine kinase promoter, which is then set to 1 in DMSO-treated groups.

### Statistical analysis

All data are displayed as the mean ± S. E. M., and *p* < 0.05 means significant difference. Data sets are analyzed by utilizing Student′s t test or one-way ANOVA, and individual group means are compared with the Student-Newman-Keuls multiple comparison test.

## Results

### IgG-IC-mediated acute lung injury is alleviated by cAMP

**Fig. 1:**
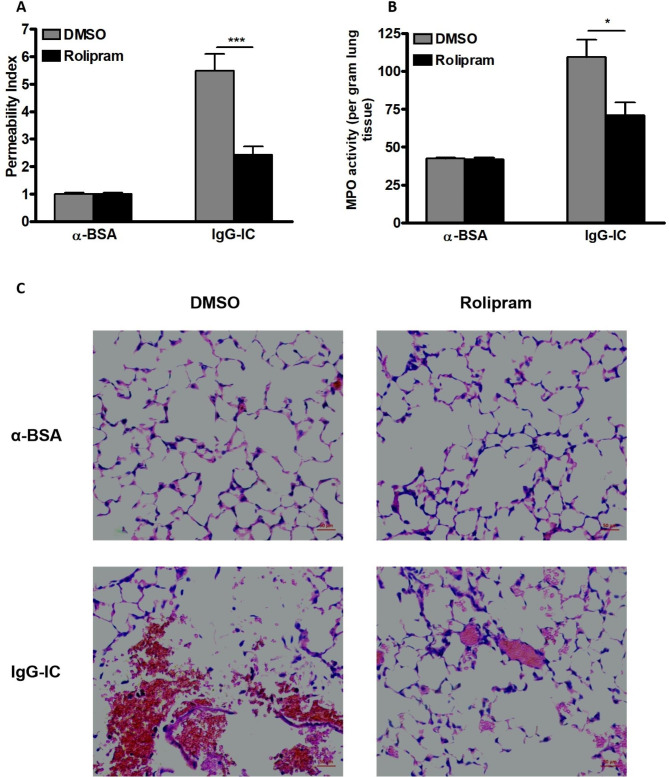
IgG-IC-induced acute lung injury is attenuated by Rolipram. Rolipram is dissolved in DMSO to obtain 10 mg/ml solution. For DMSO groups, mice are treated according to the following principle: 0.1 ml/kg. For Rolipram groups, mice are treated according to the following principle: 1 mg/kg. Mice are treated by intraperitoneal injection of DMSO + PBS or Rolipram + PBS, and the total volume is 200 µl. Then mice receive immediate airway deposition of IgG-IC. 4 h later, BAL fluids and whole lung tissues are harvested to analyze pulmonary permeability **(A)**, and MPO activity **(B)**, respectively. Data are expressed as mean ± SEM (N = 6 for control groups, and N = 10 for IgG-IC groups). * and *** suggest *p* < 0.05 and *p* < 0.001, respectively. **(C)** Mice receiving intraperitoneal treatment of DMSO or Rolipram are stimulated by airway deposition of IgG-IC. 4 h after initiation of acute lung injury, lungs are harvested and histological changes are assessed

To assess the effect of cAMP on IgG-IC-induced acute lung injury, PDE4-selective inhibitor Rolipram, which can elevate cellular cAMP concentration, is applied to the mouse model. As shown in Fig. 1A, in the presence of IgG-IC, the pulmonary permeability index is elevated more than 4.5 folds. Of note, Rolipram treatment leads to a more than 50% decrease in the tissue permeability index in IgG-IC-treated mice (Fig. 1A). Meantime, we examine the influence of Rolipram on MPO activities (an indicator reflecting neutrophil accumulation) in lungs. As expected, MPO activities in IgG-IC-treated mice double when compared with the control-treated counterparts. Interestingly, IgG-IC-triggered MPO activities in lung tissues are also significantly reduced in mice receiving Rolipram treatment. Moreover, we conduct histological examination, and find that Roliparm alone has no effect on lung architecture (Fig. 1C). However, IgG-IC-induced tissue destruction is partially reversed by Roliparm (Fig. 1C).

**Fig. 2:**
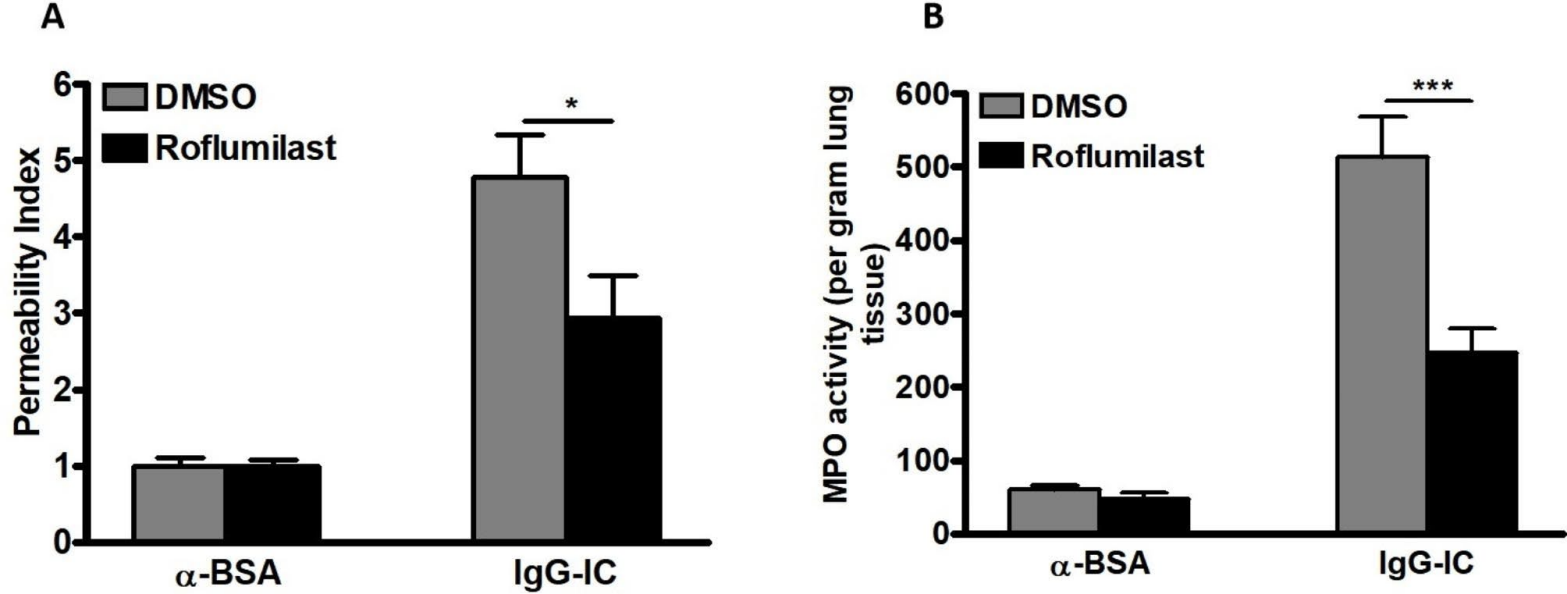
IgG-IC-induced acute lung injury is attenuated by Roflumilast. Roflumilast is dissolved in DMSO to obtain 20 mg/ml solution. For DMSO groups, mice are treated according to the following principle: 0.05 ml/kg. For Roflumilast groups, mice are treated according to the following principle: 1 mg/kg. Mice are treated by intraperitoneal injection of DMSO + PBS or Roflumilast + PBS, and the total volume is 200 µl. Then mice receive immediate airway deposition of IgG-IC. 4 h later, BAL fluids and whole lung tissues are harvested to analyze pulmonary permeability **(A)**, and MPO activity **(B)**, respectively. Data are expressed as mean ± SEM (N = 6 for control groups, and N = 10 for IgG-IC-treated groups). * and *** suggest *p* < 0.05 and *p* < 0.001, respectively

Roflumilast, another PDE4 specific inhibitor that has been used to treat COPD patients with exacerbation, is also utilized to evaluate the effect of cAMP on IgG-IC-associated acute lung injury. As shown in Fig. 2A and B, pulmonary tissue-related injury indicators including the permeability index and MPO activity are obviously amplified in mice challenged by airway deposition of IgG-IC, which are significantly reduced with application of Roflumilast. Collectively, our data demonstrate that IgG-IC-initiated acute lung injury is indeed alleviated by the cAMP whose concentration is positively regulated by the PDE4-selective inhibitors.

### Recruitment of leukocytes into alveolar spaces is reduced by cAMP

**Fig. 3:**
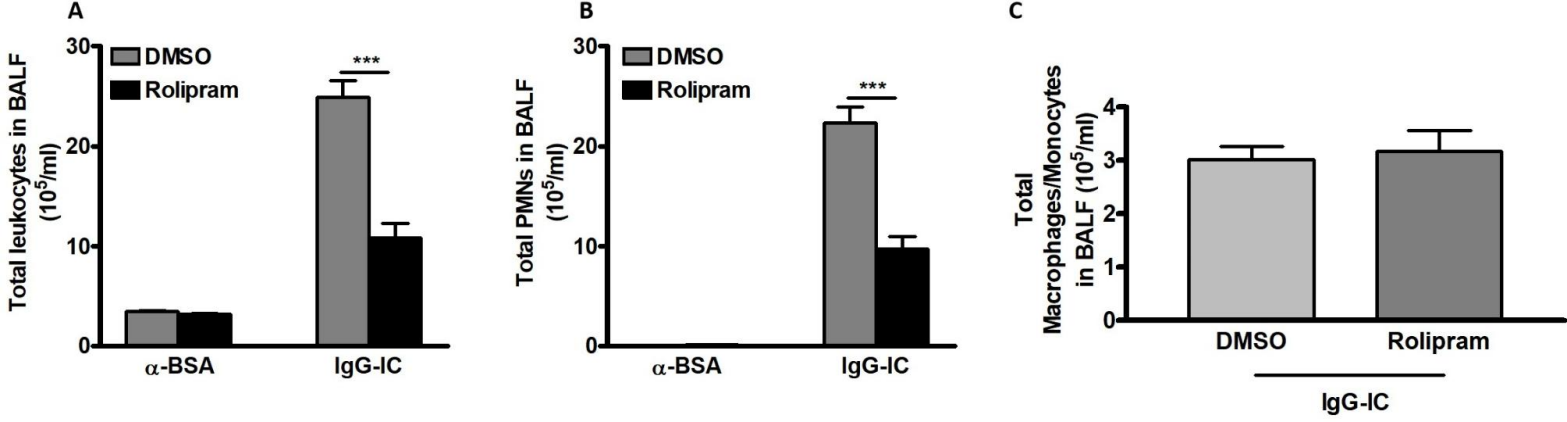
The effect of cAMP on IgG-IC-induced pulmonary recruitment of inflammatory cells. Mice are treated by intraperitoneal injection of DMSO or Rolipram (1 mg/kg), which is followed by immediate airway deposition of IgG-IC. 4 h later, BAL fluids are harvested, and total white blood cells **(A)**, neutrophils **(B)**, and macrophages/monocytes **(C)** are counted, respectively. Data are expressed as mean ± SEM (N = 6 for control groups, and N = 10 for IgG-IC-treated groups). *** suggests *p* < 0.001

Accumulation of white blood cells especially neutrophils in alveolar spaces plays an essential role in lung injury promoted by IgG-IC. Thus, we plan to elucidate the influence of cAMP on transmigration of leukocytes in IgG-IC-induced acute lung injury. We observe that in the absence of IgG-IC, leukocyte counts in BAL fluids are not altered by the PDE4 inhibitor (Fig. 3A). However, during IgG-IC-induced acute lung injury, the number of white blood cells in BAL fluids is decreased by about 60% in mice receiving peritoneal injection of Rolipram as compared with the controls (Fig. 3A). Furthermore, we investigate the role of cAMP in IgG-IC-induced transmigration of neutrophils, and find that the number of neutrophils in BAL fluids obtained from mice receiving both IgG-IC and Rolipram treatments is decreased to about 40% of the littermates treated by IgG-IC plus DMSO (Fig. 3B). However, during IgG-IC-induced acute lung injury, accumulation of macrophages/monocytes in alveolar spaces is not affected by Rolipram (Fig. 3C), which might be due to no difference in MCP-1 expression between control and Rolipram groups (Data not shown).

### IgG-IC-induced expressions of pro-inflammatory mediators are inhibited by cAMP in lungs

**Fig. 4:**
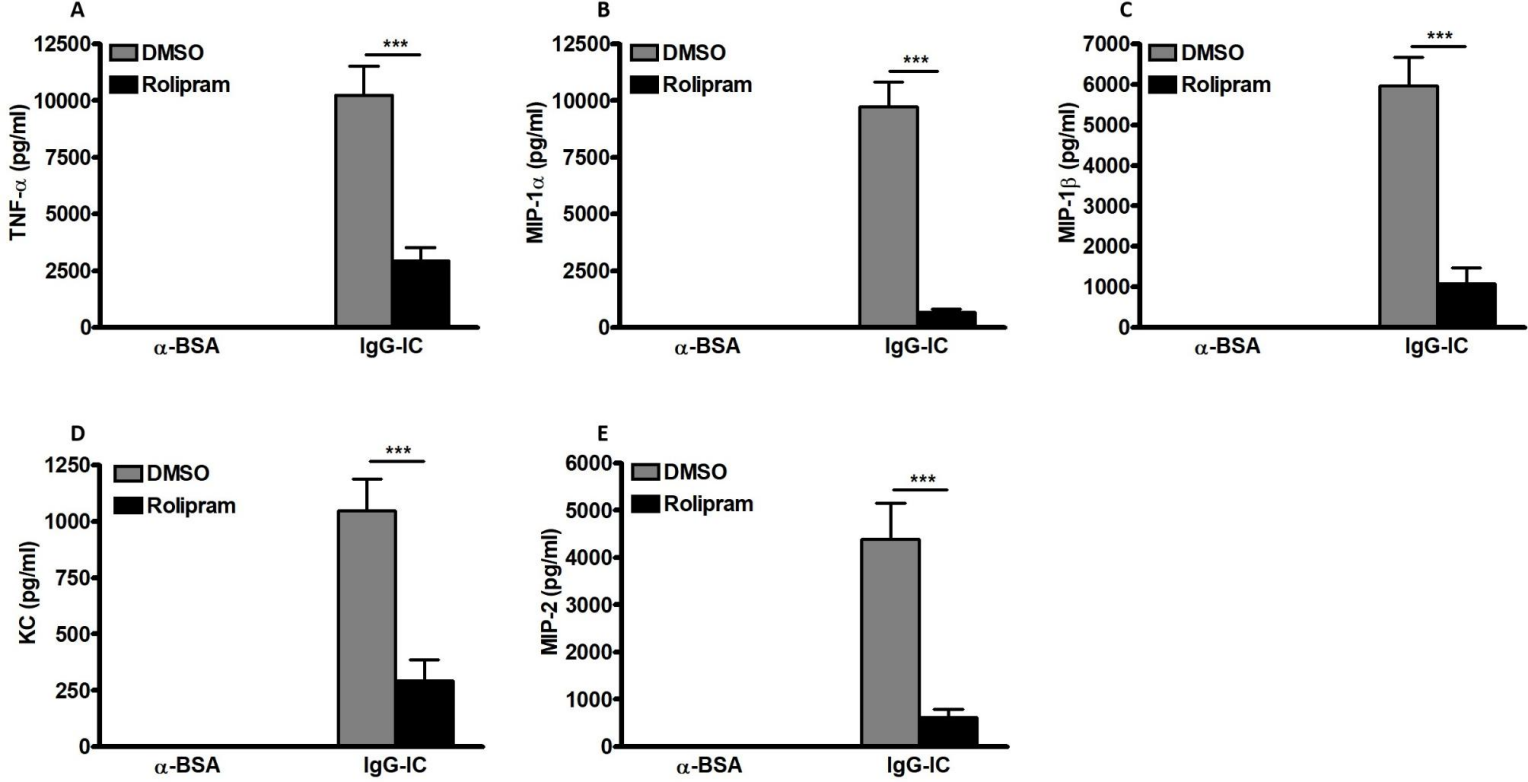
Expressions of pro-inflammatory mediators are reduced by cAMP in IgG-IC-stimulated acute lung injury. Mice are treated by intraperitoneal injection of DMSO or Rolipram (1 mg/kg), which is followed by immediate airway deposition of IgG-IC. 4 h later, BAL fluids are harvested, and levels of TNF-α **(A)**, MIP-1α **(B)**, MIP-1β **(C)**, KC **(D)**, and MIP-2 **(E)** are measured, respectively. Data are expressed as mean ± SEM (N = 6 for control groups, and N = 10 for IgG-IC groups). *** suggests *p* < 0.001

The inflammatory response is one of the critical factors involved in IgG-IC-induced acute lung injury. Thus, we attempt to dissect the influence of cAMP on expressions of pro-inflammatory mediators in IgG-IC-injured mouse lungs. As shown in Fig. 4A and E, generation of TNF-α, MIP-1α, MIP-1β, KC and MIP-2 is not detected in BAL fluids recovered from control-treated mice. However, after airway deposition of IgG-IC, levels of the above pro-inflammatory mediators are dramatically increased in BAL fluids (Fig. 4A and E). Of note, during IgG-IC-induced acute lung injury, once Rolipram is applied to the mouse model, concentrations of the cytokine and chemokines are greatly reduced in BAL fluids (Fig. 4A and E). Taken together, the above data prove that reduced productions of pro-inflammatory mediators due to peritoneal administration of Rolipram in mice may contribute to alleviation of pulmonary tissue injury stimulated by airway deposition of IgG-IC.

### IgG-IC-induced expressions of pro-inflammatory mediators are inhibited by cAMP in in vitro experiments

**Fig. 5:**
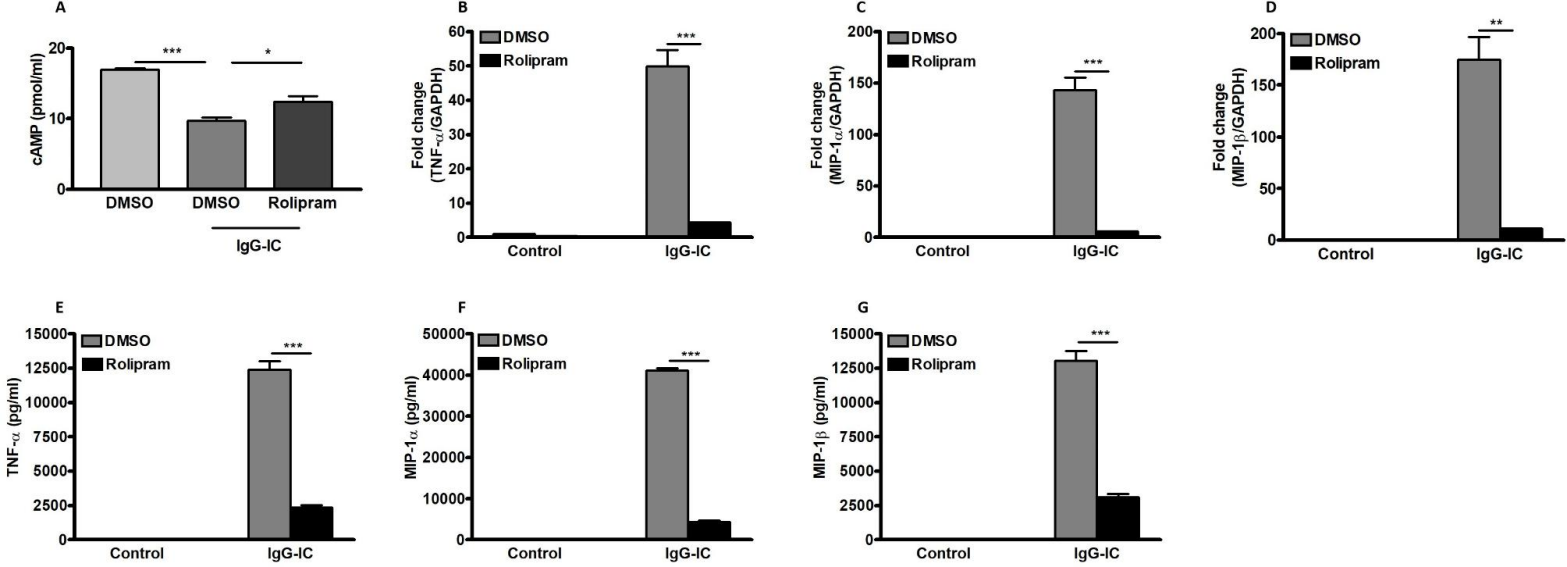
Expressions of pro-inflammatory mediators are reduced by cAMP in IgG-IC-treated macrophages. Rolipram is dissolved in DMSO to obtain 10 mM solution. RAW264.7 cells are pre-treated with DMSO (1:1000 dilution) or Rolipram (10 µM) for 1 h. Then the cells are treated with or without 100 µg/ml of IgG-IC. 3 h later, the cells are lysed and intracellular cAMP concentrations are measured **(A)**. Total cellular RNAs and supernatants are harvested at 3 and 6 h time points, respectively. Then expressions of TNF-α (B and E), MIP-1α (**C** and **F**), and MIP-1β (**D** and **G**) are examined at RNA and protein levels, respectively. Data are expressed as mean ± SEM (N = 3 for qPCR, and N = 6 for ELISA). *, ** and *** suggest *p* < 0.05, 0.01 and 0.01, respectively

During IgG-IC-induced acute lung injury, macrophages are initiators of excessive inflammatory responses through secretion of a variety of pro-inflammatory mediators. Thus, macrophages incubated with IgG-IC are used to mimic the in vivo experiments. Firstly, we investigate the influence of IgG-IC on intracellular cAMP level, and observe that cAMP concentration is significantly reduced by IgG-IC stimulation, which is partially reversed by Rolipram treatment (Fig. 5A). Then the effect of cAMP on IgG-IC-stimulated expressions of pro-inflammatory mediators is clarified. As shown in Fig. 5B and D, mRNA levels of TNF-α, MIP-1α and MIP-1β are increased by 48.9, 142.1 and 173.5 folds, respectively in macrophages treated by IgG-IC when compared with those of the controls. However, in the presence of Rolipram, IgG-IC-mediated expressions of TNF-α, MIP-1α and MIP-1β mRNAs are reduced by 91%, 96% and 93%, respectively (Fig. 5B and D). Proteins are direct executors of cell functions. Thus, the influence of cAMP on IgG-IC-stimulated expressions of TNF-α, MIP-1α and MIP-1β is further investigated at protein level. We observe that secretion of TNF-α, MIP-1α and MIP-1β is dramatically elevated by macrophages incubated with IgG-IC (Fig. 5E and G). Of note, Rolipram treatment leads to a significant decrease in IgG-IC-mediated expressions of TNF-α, MIP-1α and MIP-1β at protein level (Fig. 5E and G).

**Fig. 6:**
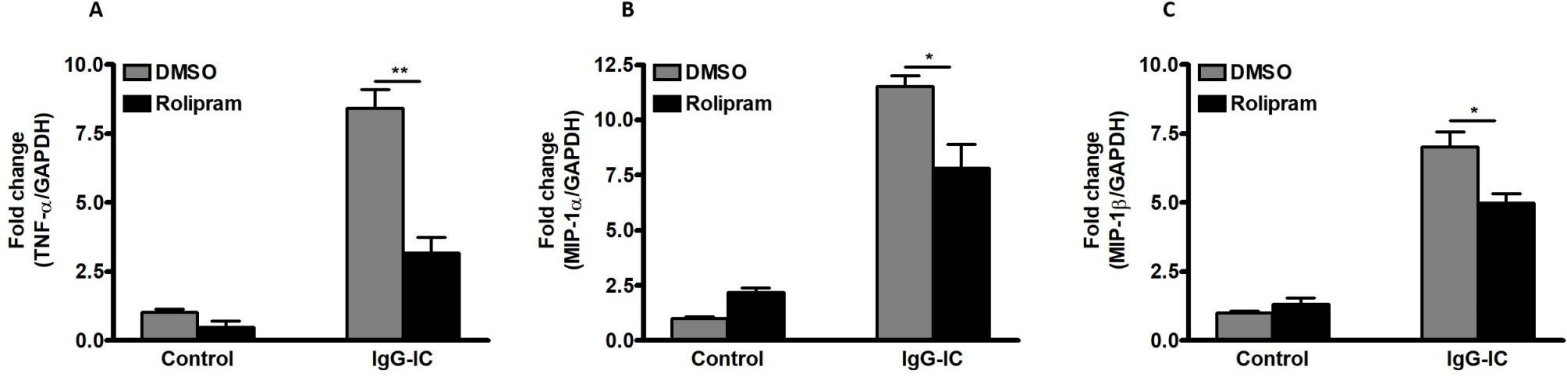
Expressions of pro-inflammatory mediators are reduced by cAMP in IgG-IC-treated neutrophils. The mouse receives intraperitoneal injection of 3% of Thioglycollate (BD, USA) dissolved in ddH_2_O. Four hours later, neutrophils are harvested. Neutrophils are pre-treated with DMSO or Rolipram (10 µM) for 1 h. Then the cells are treated with or without 100 µg/ml of IgG-IC. 3 h later, total cellular RNAs are isolated. Then expressions of TNF-α **(A)**, MIP-1α **(B)**, and MIP-1β **(C)** are examined. Data are expressed as mean ± SEM (N = 3). * and ** suggest *p* < 0.05 and 0.01, respectively

During IgG-IC-induced acute pulmonary inflammation, neutrophils are recruited into lung tissues, and could play vital roles in tissue damage by producing kinds of pro-inflammatory mediators. Therefore, we also explore the effect of Rolipram on IgG-IC-induced inflammation in neutrophils, and find that IgG-IC-mediated expressions of TNF-α, MIP-1α and MIP-1β are all significantly reduced in neutrophils treated by Rolipram (Fig. 6A C).

### PKA is involved in cAMP-mediated negative regulation of IgG-IC-induced expressions of pro-inflammatory mediators and acute lung injury

**Fig. 7:**
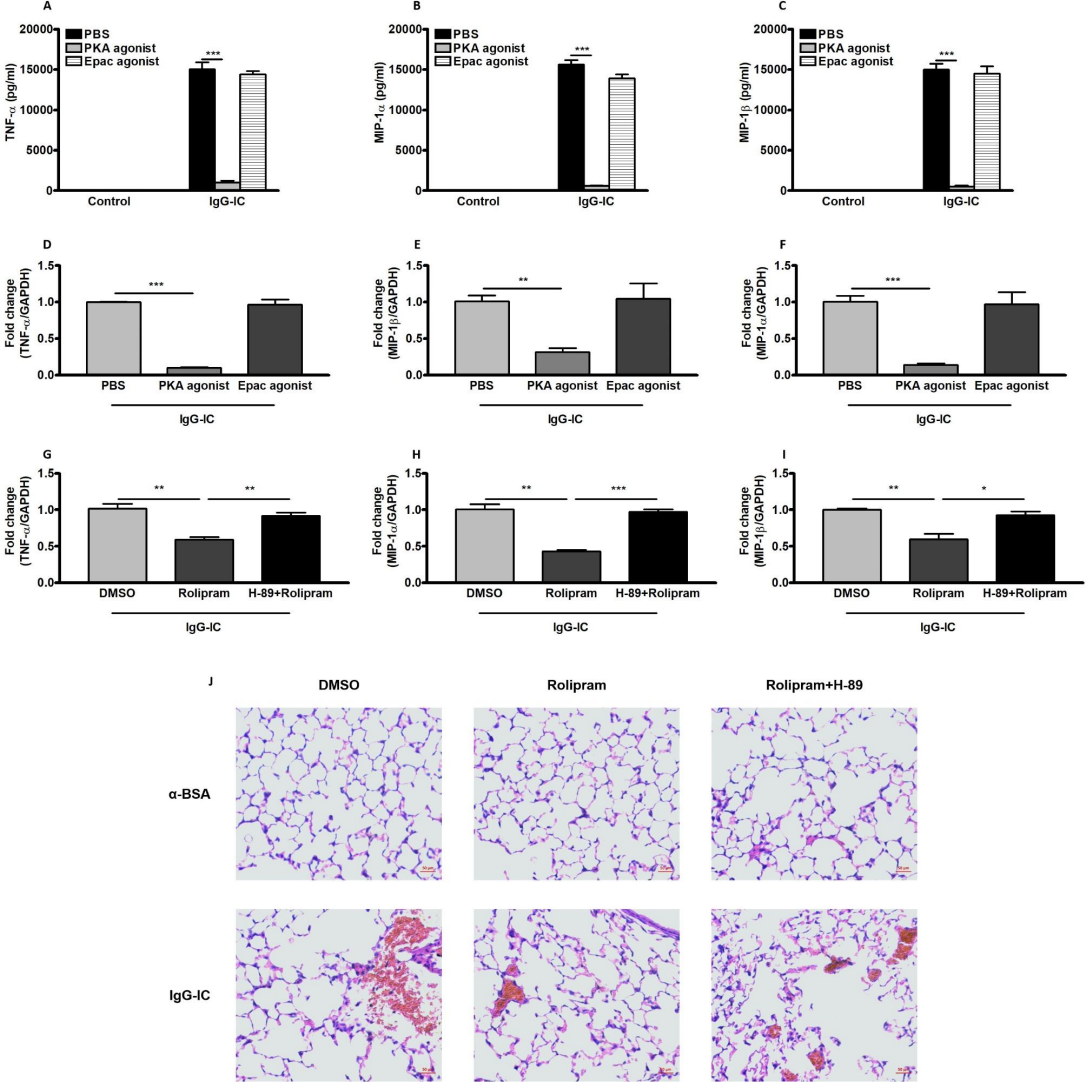
PKA is involved in cAMP-mediated negative regulation of IgG-IC-induced expressions of pro-inflammatory mediators and acute lung injury. RAW264.7 cells are pre-treated with 2 mM of PBS/PKA agonist/Epac agonist for 30 min. Then the cells are treated with or without 100 µg/ml of IgG-IC. Cell-free supernatants are harvested 6 h later. ELISA is used to measure expressions of TNF-α **(A)**, MIP-1α **(B)**, and MIP-1β **(C)**. RAW264.7 cells are pre-treated with 0.2 mM of PBS/PKA agonist/Epac agonist for 30 min. Then the cells are treated with or without 100 µg/ml of IgG-IC. RNAs are harvested 3 h later. qPCR is used to measure expressions of TNF-α **(D)**, MIP-1α **(E)**, and MIP-1β **(F)**. H-89 is dissolved in DMSO to obtain 10 mM solution. RAW264.7 cells are pre-treated with DMSO (1:500 dilution), Rolipram (10 µM) or H-89 (10 µM) + Rolipram (10 µM) for 1 h. Then the cells are treated with or without 100 µg/ml of IgG-IC. RNAs are harvested 3 h later. qPCR is used to measure expressions of TNF-α **(G)**, MIP-1α **(H)**, and MIP-1β **(I)**. J. Rolipram and H-89 are dissolved in DMSO to obtain 10 mg/ml, and 25 mg/ml solutions, respectively. For DMSO group, mice are treated according to the following principle: 0.5 ml/kg. For Rolipram group, mice are treated according to the following principle: 1 mg/kg Rolipram + 0.4 ml/kg DMSO. For Rolipram + H-89 group, mice are treated according to the following principle: 1 mg/kg Rolipram + 10 mg/kg H-89. Mice are treated by intraperitoneal injection of DMSO, Rolipram or H-89 + Rolipram, which is followed by airway deposition of IgG-IC. 4 h later, lungs are harvested and histological changes are assessed. Data are expressed as mean ± SEM (N = 6 for ELISA, and N = 3 for qPCR). *, ** and *** suggest *p* < 0.05, 0.01 and 0.01, respectively

Epac and PKA are two critical downstream targets of cAMP, and previous study has demonstrated that Epac but not PKA inhibits inflammatory responses in LPS-treated macrophages and lungs [18]. Here, we also clarify their effects on IgG-IC-stimulated inflammation in macrophages and lungs. As shown in Fig. 7A C, productions of TNF-α, MIP-1α and MIP-1β are hardly measured in control-treated macrophages. However, once upon IgG-IC stimulation, secretion of the above mentioned pro-inflammatory mediators by macrophages are dramatically elevated (Fig. 7A C). Of note, PKA agonist treatment results in a more than 90% decrease in TNF-α, MIP-1α and MIP-1β expressions (Fig. 7A C). Of interest, IgG-IC-induced expressions of the pro-inflammatory mediators are almost not affected by Epac agonist (Fig. 7A C). Moreover, we examine the effect of lower concentration of PKA and Epac agonists on IgG-IC-induced inflammation in macrophages, and observe similar results as demonstrated above (Fig. 7D F).

We further elucidate whether cAMP exerts its anti-inflammatory effect through PKA, and observe that in the presence of Rolipram, IgG-IC-stimulated expressions of TNF-α, MIP-1α and MIP-1β are obviously reduced, which is almost completely reserved by PKA inhibitor H-89 (Fig. 7G and I). Furthermore, in vivo data show that inhibitory effect of Rolipram on IgG-IC-induced acute lung injury is blocked with the application of PKA inhibitor H-89 (Fig. 7J). Thus, cAMP downstream molecule PKA but not Epac is the negative regulator of IgG-IC-induced expressions of pro-inflammatory mediators and acute lung injury.

### IgG-IC-mediated activation of MAPKs is suppressed by cAMP-PKA signal

**Fig. 8:**
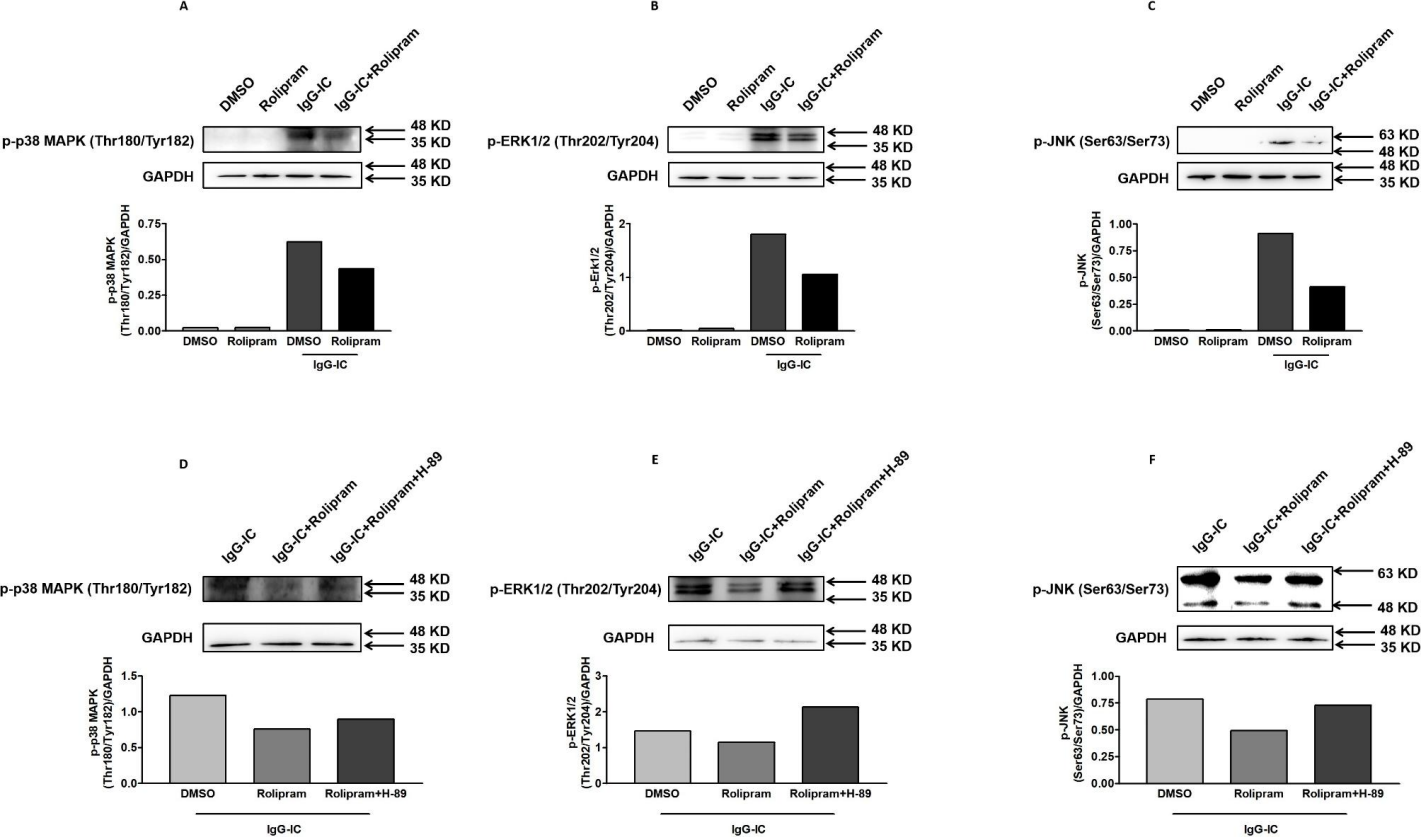
Phosphorylation of MAPKs is negatively regulated by cAMP-PKA signal in IgG-IC-treated macrophages. RAW264.7 cells are pre-treated with DMSO or Rolipram (10 µM) for 1 h. Then the cells are treated with or without 100 µg/ml of IgG-IC. RAW264.7 cells are pre-treated with DMSO, Rolipram (10 µM) or Rolipram (10 µM) + H-89 (10 µM) for 1 h. Then the cells are treated with or without 100 µg/ml of IgG-IC. Whole cellular proteins are extracted 30 min later. Activation of p38 MAPK (**A** and **D**), ERK1/2 (B and E), and JNK (**C** and **F**) is investigated by Western blot assays, respectively

Our previous data have demonstrated that MAPKs are involved in regulation of pro-inflammatory mediators′ expressions [30–32]. So, we tend to examine if cAMP-PKA signal exerts its anti-inflammatory effect through MAPK pathway. As shown in Fig. 8A-C, in the presence of IgG-IC, phosphorylation of p38 MAPK, ERK1/2 and JNK is increased, while the IgG-IC-induced activities of MAPK family members are inhibited by Rolipram that can increase cAMP level in cells. We further investigate whether cAMP regulates MAPKs phosphorylation through PKA. As shown in Fig. 8D-F, the regulatory role of cAMP in phosphorylation of p38 MAPK, ERK1/2 and JNK is suppressed by PKA inhibitor H-89 in IgG-IC-treated macrophages.

### AP-1- and C/EBP-mediated transcriptions are inhibited by cAMP

**Fig. 9:**
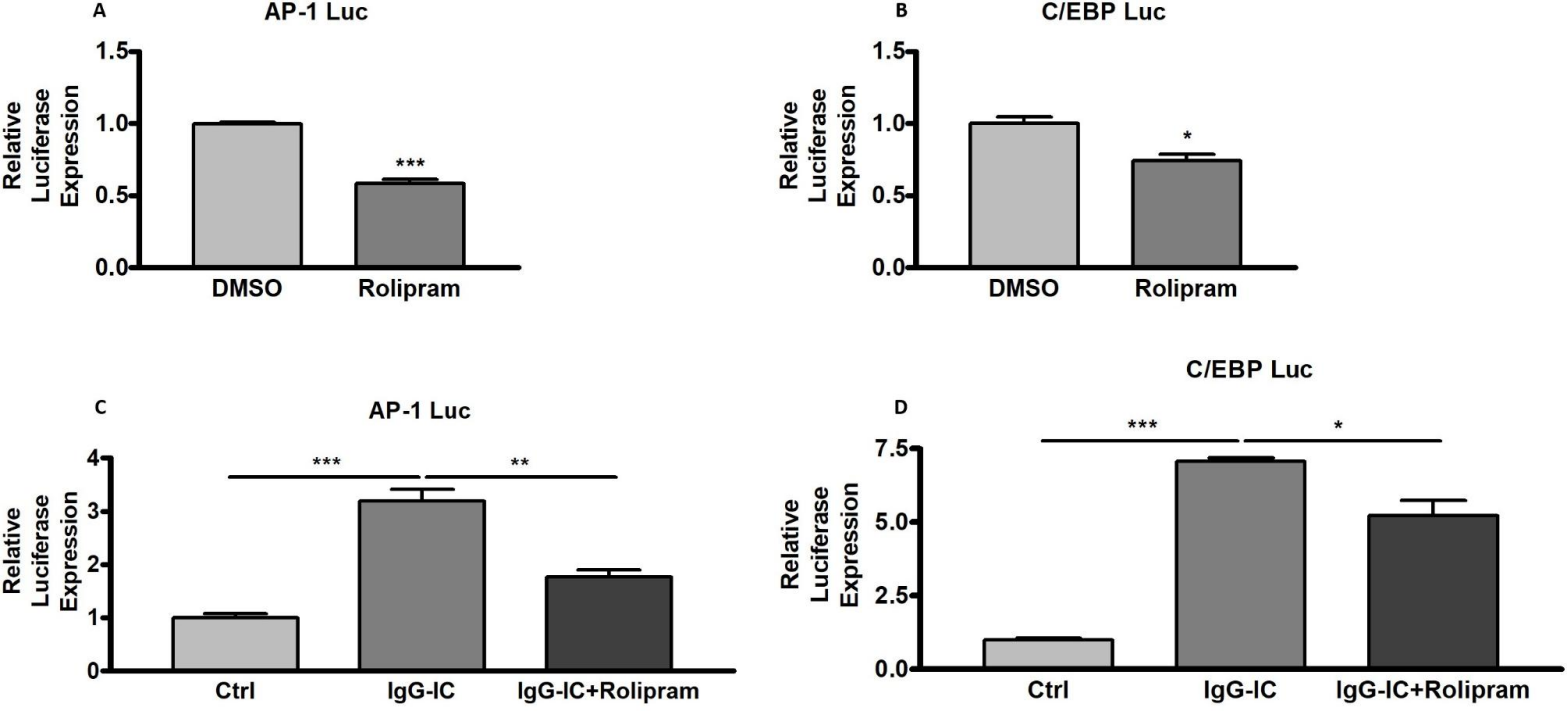
AP-1- and C/EBP-mediated transcriptions are reduced by cAMP. HEK293/RAW264.7 cells are transfected with the indicated plasmids. 24 h later, HEK293 cells are treated with DMSO or Rolipram (10 µM) for 6 h. RAW264.7 cells are pre-treated with DMSO or Rolipram for 1 h. Then the cells are treated with or without 100 µg/ml of IgG-IC for 6 h. The cells are lysed, and subjected to luciferase assays. Data are expressed as mean ± SEM (N = 3). *, ** and *** suggest *p* < 0.05, 0.01 and 0.01, respectively

The above result has proved that dephosphorylation of JNK is promoted by cAMP. In addition, the regulatory role of JNK in AP-1-mediated gene transcription has been widely demonstrated. Thus, we further investigate whether cAMP affects transcription activity of AP-1 which plays essential roles in a variety of pro-inflammatory mediators′ transcriptions. As shown in Fig. 9A, Rolipram treatment leads to a 41% decrease in AP-1 activity. Our published paper has shown that transcription activity mediated by C/EBP is positively regulated by p38 MAPK and ERK1/2 [30]. We speculate that cAMP-induced dephosphorylation of p38 MAPK and ERK1/2 may result in a decrease in C/EBP activity. Therefore, we perform luciferase assay to evaluate the influence of cAMP on C/EBP-mediated transcription, and find that C/EBP activity is reduced to 74% of the control by Rolipram (Fig. 9B). The above data are obtained from HEK293 cells, we further confirm the conclusions in macrophages, and observe that IgG-IC-stimulated AP-1 and C/EBP activities are reduced by Rolipram (Fig. 9C and D).

## Discussion

Acute lung injury and acute respiratory distress syndrome are serious pulmonary diseases with high rates of mortality and morbidity. In the United States, the incidence of ALI and ARDS in patients older than 15 is 78.9 and 58.7 cases/100,000 individuals per year, respectively, and the overall mortality rate of ALI and ARDS remains as high as 38.5% and 41.1%, respectively, which is attributable to be lack of valid therapeutic methods [33]. Therefore, it is urgently needed to develop novel treatments based on the mechanisms of the disease. ALI is characterized by disruption of alveolar-capillary membrane integrity, which is accompanied by recruitment of white blood cells especially neutrophils into the alveoli [34, 35]. Of note, pro-inflammatory mediators generated during ALI are the primary mechanisms of pulmonary histopathological changes. Thus, prevention from excessive inflammatory responses might pave the way for treating the disease.

cAMP, one of the second messenger molecules, could modify the functions of various innate immune cells including macrophages through inhibiting production of pro-inflammatory mediators [36]. Cellular levels of cAMP are mainly determined by the activities of adenylyl cyclases (AC) and PDE4 [37, 38]. Adenylyl cyclases enhance conversion of ATP to cAMP, while PDE4 family members increase hydrolysis of cAMP to AMP and, therefore, cellular cAMP concentrations could be amplified by activating AC or silencing PDE4. In the current study, elevation of cellular cAMP concentrations is achieved by application of PDE4 specific inhibitors. We find that IgG-IC-induced expressions of pro-inflammatory mediators in macrophages and lungs are reduced with an increase of cAMP level. PDE4 family is composed of four members: PDE4A, PDE4B, PDE4C and PDE4D [39]. Except for PDE4C, other three family members are highly expressed in macrophages. The accurate roles of PDE4A, PDE4B and PDE4D in IgG-IC-triggered inflammation are open questions, and the answers to the questions will be conducive to development of an inhibitor targeting the specific member, thus reducing unnecessary side effects produced by broad-spectrum inhibitors. Macrophages, which belong to the innate immune system, play pivotal roles in acute lung injury. There are increasing data showing that depletion of macrophages attenuates ALI through down-regulating generation of pro-inflammatory mediators, including cytokines and chemokines and, therefore, controlling expressions of the inflammatory factors derived from macrophages might contribute to resolution of the tissue damage [40, 41]. Thus, development of the inhibitor targeting specific PDE4 family member in macrophages might be more suitable for the needs of precision medicine projects around the world.

Epac and PKA are two critical downstream targets of cAMP. Previous study has demonstrated that Epac but not PKA inhibits inflammatory responses in LPS-treated macrophages [18]. In this paper, we also examine their effects on IgG-IC-mediated generation of pro-inflammatory mediators. To our surprise, PKA but not Epac is involved in cAMP-mediated negative regulation of expressions of cytokine and chemokines upon IgG-IC stimulation. Therefore, selection of downstream target by cAMP is dependent on properties of certain inflammatory stimulants.

## Conclusion

In conclusion, our data demonstrate that IgG-IC-induced acute lung injury is alleviated with application of the PDE4-selective inhibitor, which could increase cellular cAMP level. Furthermore, we find that cAMP attenuates lung tissue damages by reducing expressions of pro-inflammatory mediators. Mechanistically, cAMP activates PKA, leading to dephosphorylation of activated p38 MAPK, ERK1/2 and JNK, resulting in decreased C/EBP- and AP-1-mediated transcriptions that promote pro-inflammatory mediators′ expressions. Collectively, our data provide experimental evidences for potential application of PDE4 specific inhibitor to clinic for treatment of IgG-IC-related acute lung injury.

### Electronic supplementary material

Below is the link to the electronic supplementary material.

Supplementary Material 1

## Data Availability

All the data and material-related information in the current paper are available from the authors upon reasonable request.

## Abbreviations

ALI: Acute lung injury
ARDS: Acute respiratory distress syndrome
PDE4: Phosphodiesterase-4
cAMP: Cyclic adenosine 3′, 5′-monophosphate
IgG-IC: IgG immune complex
PKA: Protein kinase A
Epac: Exchange proteins directly activated by cAMP
C/EBP: CCAAT/enhancer binding protein
AP-1: Activation protein 1
JNK: C-Jun N-terminal kinase
AC: Adenylyl cyclases
COPD: Chronicobstructive pulmonary disease
FBS: Fetal bovine serum
BSA: Bovine serum albumin
α-BSA: Anti-BSA antibody
DMSO: Dimethyl sulfoxide
TNF-α: Tumor necrosis factor alpha
MIP-1α: Macrophage inflammatory protein 1 alpha
MIP-1β: Macrophage inflammatory protein 1 beta
MIP-2: Macrophage inflammatory protein 2
KC: Keratinocyte-derived cytokine
MPO: Myeloperoxidase
BAL: Bronchoalveolar lavage
H&E: Haematoxylin and eosin
BCA: Bicinchoninic acid
