# Supplementary material for: IgG immune complex-induced acute lung injury is ameliorated by cAMP via down-regulation of C/EBP- and AP-1-mediated transcriptions
Source: J Inflamm (Lond). 2023 Oct 20;20:34. doi: 10.1186/s12950-023-00359-6 (PMC10588139; doi:10.1186/s12950-023-00359-6)
Supplement: Supplementary file 1 — Supplementary Material 1 [file 12950_2023_359_MOESM1_ESM.docx]

**Figure 8A**

Lane 1 2 3 4


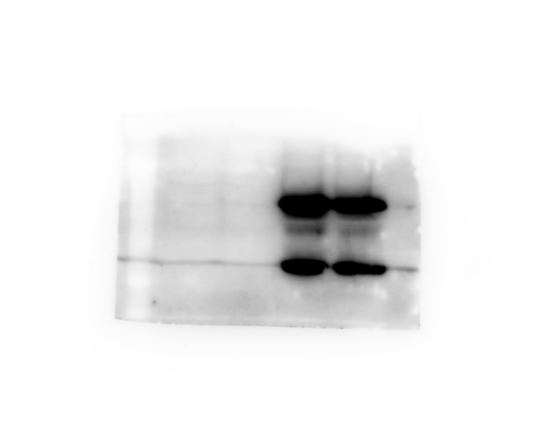


Lane 1 2 3 4


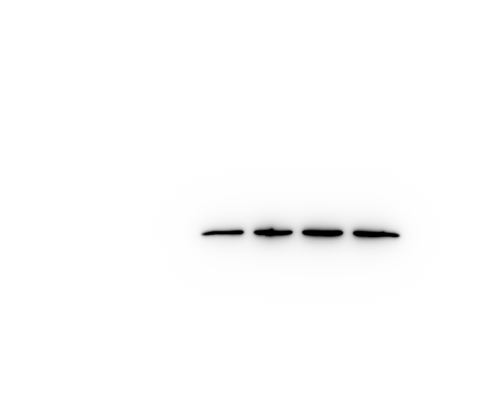


Lane1: DMSO

Lane 2: Rolipram

Lane3: IgG-IC

Lane4: IgG-IC+Rolipram

**Figure 8B**

Lane 1 2 3 4


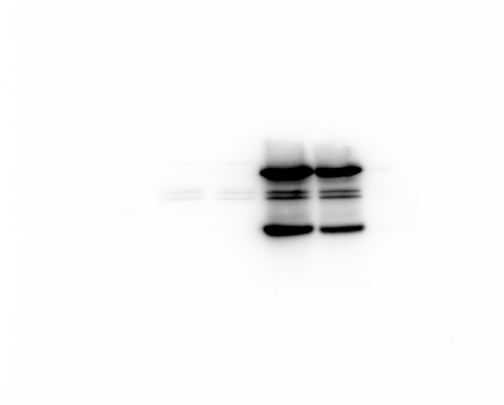


Lane 1 2 3 4


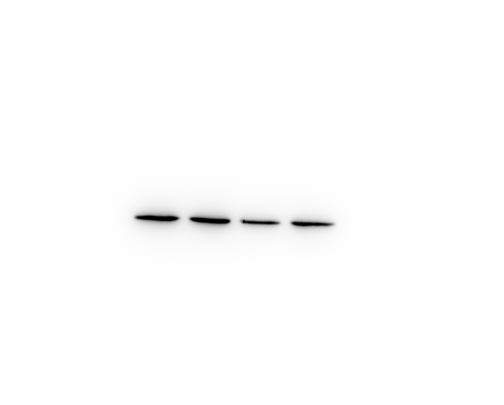


Lane1: DMSO

Lane 2: Rolipram

Lane3: IgG-IC

Lane4: IgG-IC+Rolipram

**Figure 8C**

Lane 1 2 3 4

**
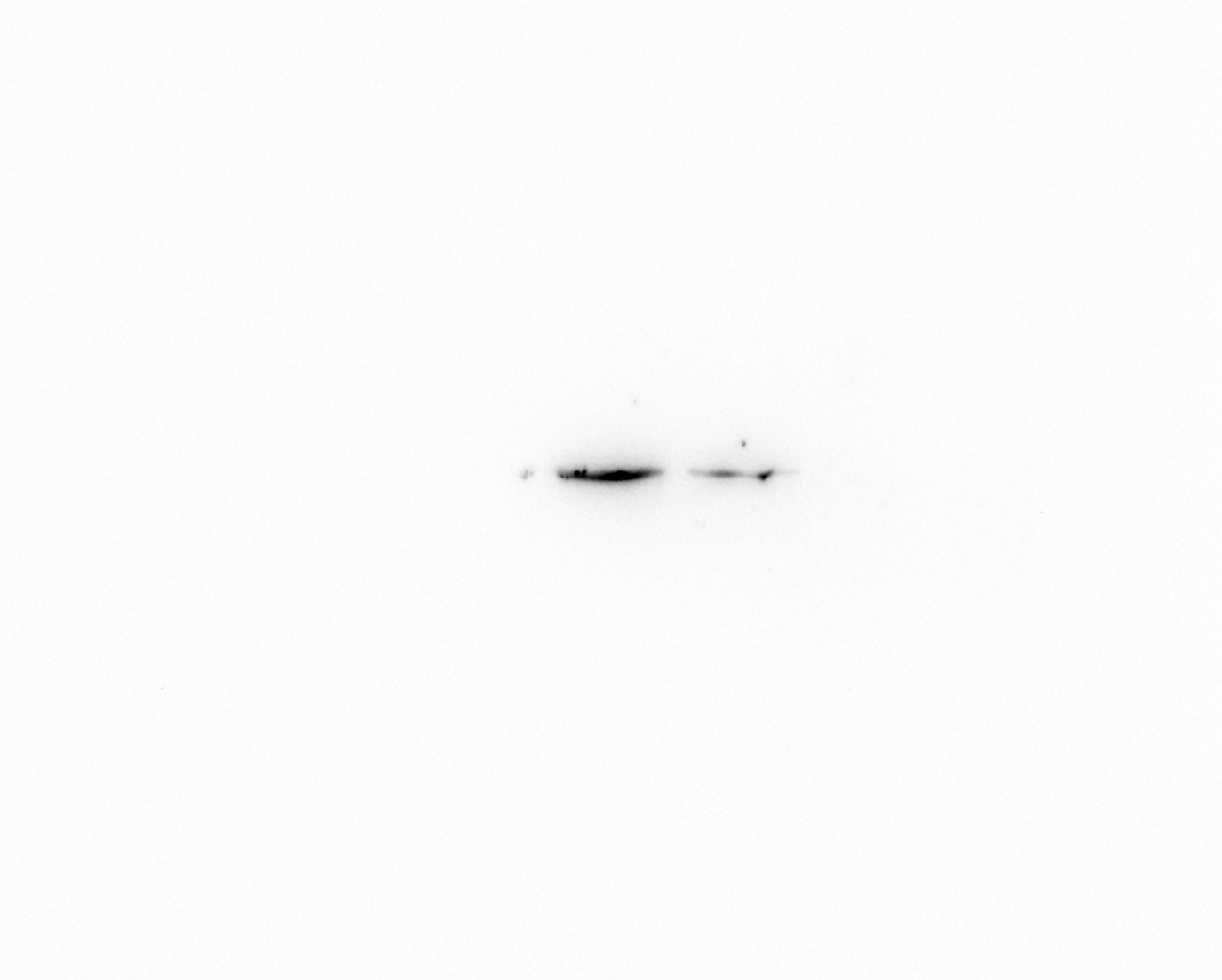
**

Lane 1 2 3 4


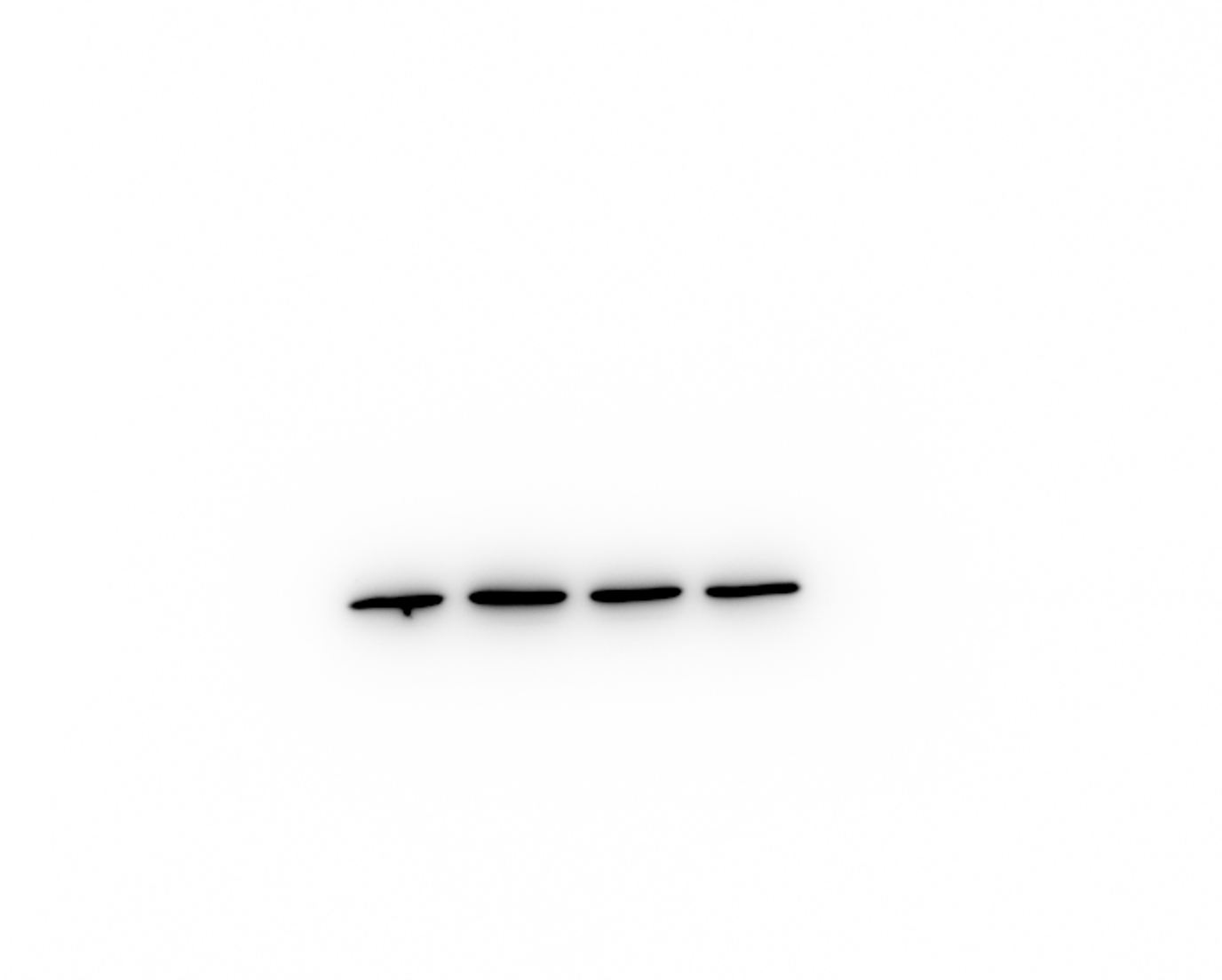


Lane1: DMSO

Lane 2: Rolipram

Lane3: IgG-IC

Lane4: IgG-IC+Rolipram

**Figure 8D**

Lane 1 2 3


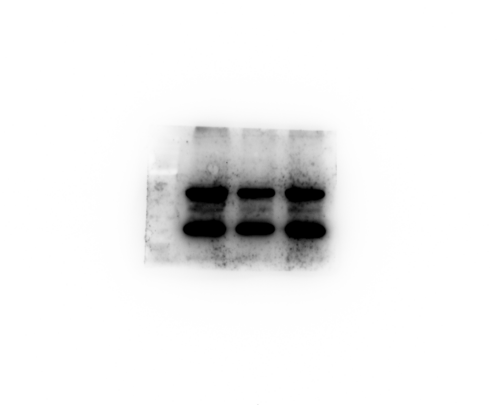


Lane 1 2 3


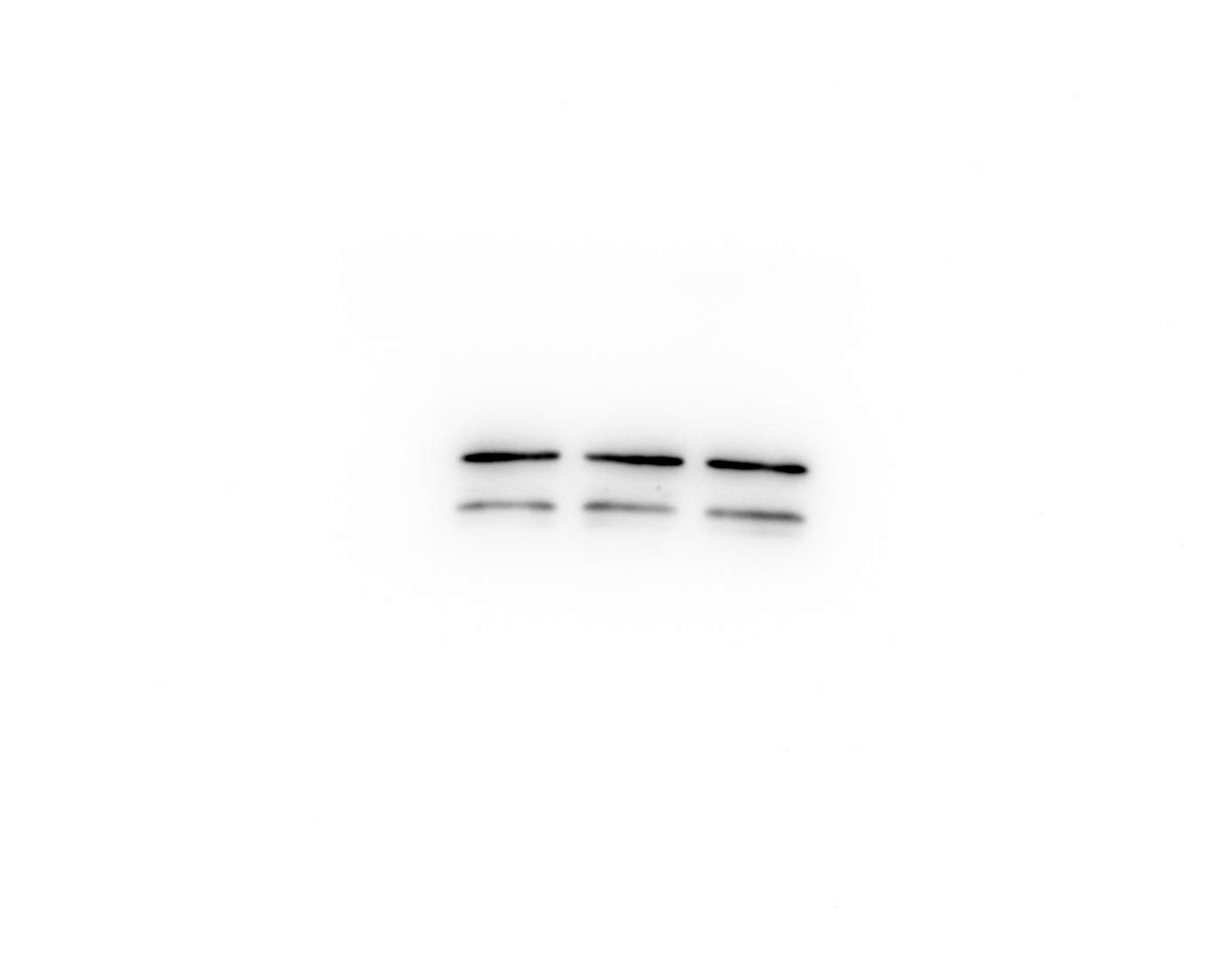


Lane 1: IgG-IC

Lane 2: IgG-IC+Rolipram

Lane 3: IgG-IC+Rolipram

**Figure 8E**

Lane 1 2 3


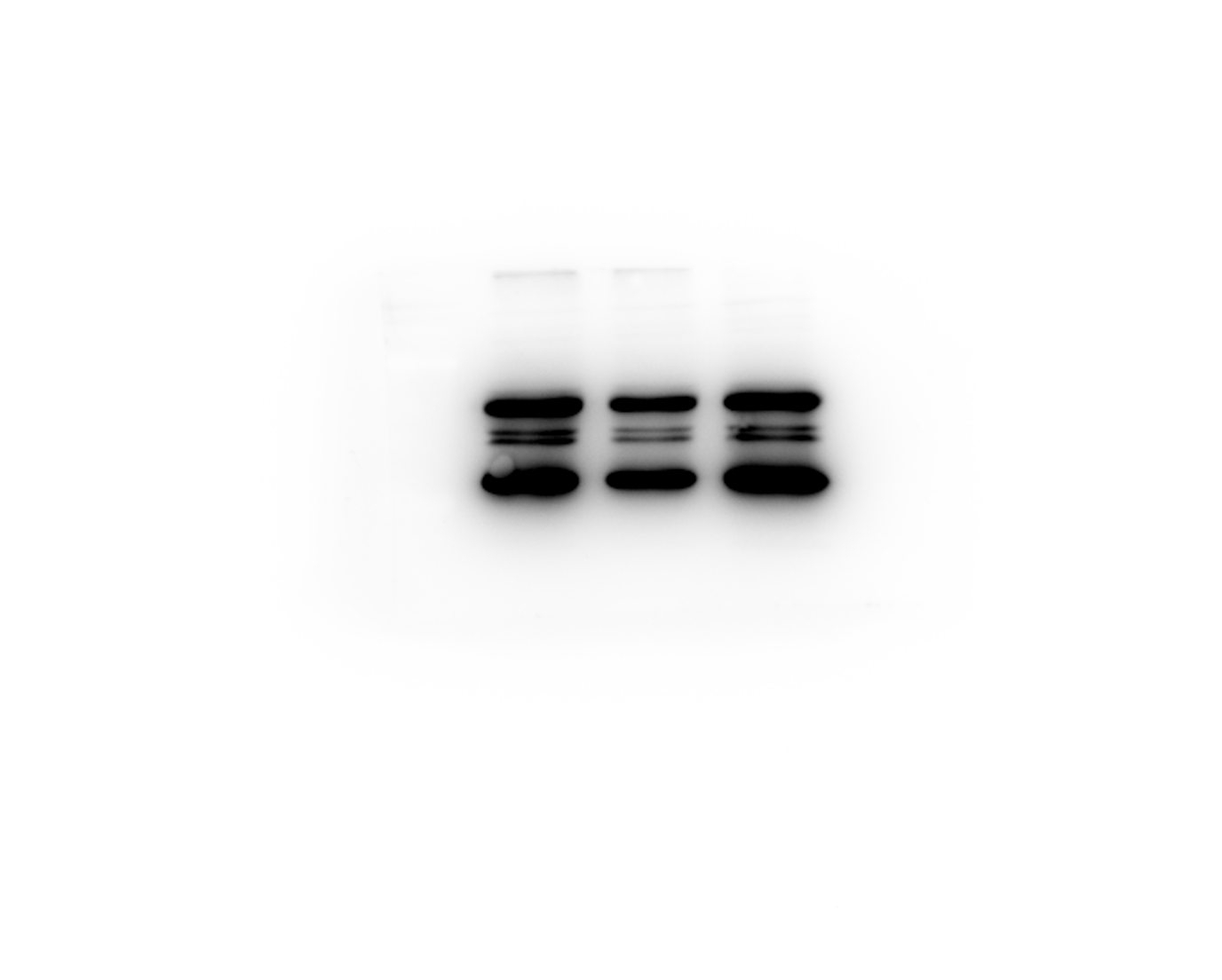


Lane 1 2 3


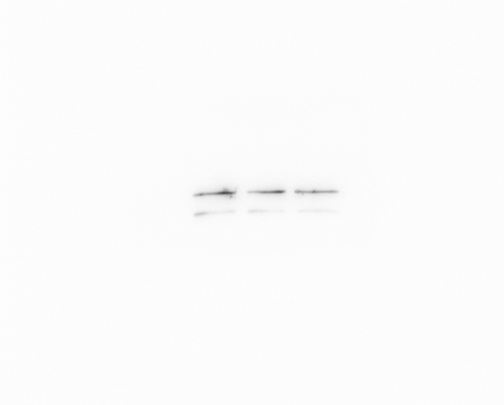


Lane 1: IgG-IC

Lane 2: IgG-IC+Rolipram

Lane 3: IgG-IC+Rolipram

**Figure 8F**

Lane 1 2 3


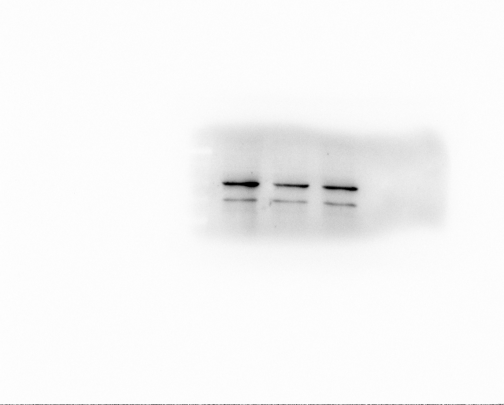


Lane 1 2 3


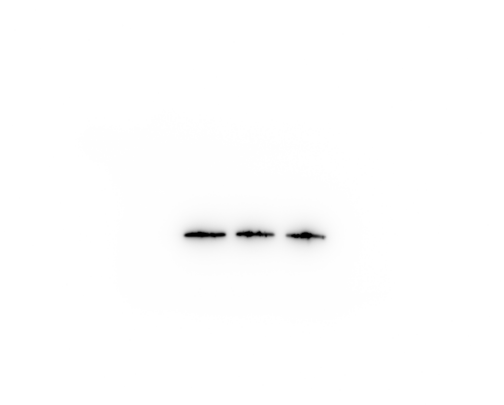


Lane 1: IgG-IC

Lane 2: IgG-IC+Rolipram

Lane 3: IgG-IC+Rolipram

**Fig. 8 Phosphorylation of MAPKs is negatively regulated by cAMP-PKA signal in IgG-IC-treated macrophages.** RAW264.7 cells are pre-treated with DMSO or Rolipram (10 μM) for 1 h, which is followed by treatment with 100 μg/ml of IgG-IC. RAW264.7 cells are pre-treated with DMSO, Rolipram (10 μM) or Rolipram (10 μM)+H-89 (10 μM) for 1 h, which is followed by treatment with 100 μg/ml of IgG-IC. Whole cellular proteins are extracted 30 min later. Then activation of p38 MAPK (A and D), ERK1/2 (B and E), and JNK (C and F) is investigated by Western blot assays, respectively.
